# Abnormal﻿ composition of microbiota in the gut and skin of imiquimod-treated mice

**DOI:** 10.1038/s41598-021-90480-4

**Published:** 2021-05-28

**Authors:** Hiroyo Shinno-Hashimoto, Yaeko Hashimoto, Yan Wei, Lijia Chang, Yuko Fujita, Tamaki Ishima, Hiroyuki Matsue, Kenji Hashimoto

**Affiliations:** 1 Department of Dermatology, Chiba University Graduate School of Medicine, Chiba, 260-8670 Japan; 2 Division of Clinical Neuroscience, Chiba University Center for Forensic Mental Health, Chiba, 260-8670 Japan; 3 Department of Respirology, Chiba University Graduate School of Medicine, Chiba, 260-8670 Japan; 4 Key Laboratory of Medical Electrophysiology of Ministry of Education and Medical Electrophysiological Key Laboratory of Sichuan Province, Collaborative Innovation Center for Prevention and Treatment of Cardiovascular Disease, Institute of Cardiovascular Research, Southwest Medical University, Luzhou, 646000 Sichuan China

**Keywords:** Skin diseases, Depression, Bacterial host response, Microbiology

## Abstract

Psoriasis is a chronic, inflammatory skin disease. Although the precise etiology of psoriasis remains unclear, gut–microbiota axis might play a role in the pathogenesis of the disease. Here we investigated whether the composition of microbiota in the intestine and skin is altered in the imiquimod (IMQ)-treated mouse model of psoriasis. Topical application of IMQ to back skin caused significant changes in the composition of microbiota in the intestine and skin of IMQ-treated mice compared to control mice. The LEfSe algorithm identified the species *Staphylococcus lentus* as potential skin microbial marker for IMQ group. Furthermore, there were correlations for several microbes between the intestine and skin, suggesting a role of skin–gut–microbiota in IMQ-treated mice. Levels of succinic acid and lactic acid in feces from IMQ-treated mice were significantly higher than control mice. Moreover, the predictive functional analysis of the microbiota in the intestine and skin showed that IMQ caused alterations in several KEGG pathways. In conclusion, the current data indicated that topical application with IMQ to skin alters the composition of the microbiota in the gut and skin of host. It is likely that skin–gut microbiota axis plays a role in pathogenesis of psoriasis.

## Introduction

Psoriasis is a common chronic inflammatory disease of skin. A recent systematic review reports that the prevalence of psoriasis in children and adults ranged from 0 to 1.37% and 0.51 to 11.43%, respectively^1^. Furthermore, psoriasis is associated strongly with depression, cardiovascular disease, diabetes, and metabolic syndrome, which influence quality of life in patients^2–6^. Although excessive activation of the immune system plays a major role in the pathogenesis of psoriasis, the precise pathogenesis of this disease remains unclear^3,6^.

Accumulating evidence suggests the physiological and metabolic roles of the microbiota in human health and diseases^7–13^. It is well known that microbes are colonized in the gastrointestinal tract, skin, oral mucosa, vagina and airway in the human, and that the colon is the most abundant site for microbes, followed by the skin^14^. Altered composition of gut microbiota in patients with psoriasis has been reported^15–19^. Furthermore, there are also several reports showing abnormal composition of skin microbiota in patients with psoriasis^20–25^. These findings suggest that alterations in the microbiome in the intestine and on the skin might play a role in the pathogenesis of psoriasis, and that improvement of altered composition of microbiota could be a therapeutic approach for this disease^14^.

Imiquimod (IMQ), a Toll-like receptor 7 agonist, has been used widely as mouse model of psoriasis to understand the inflammatory responses of the skin^26,27^. It is also reported that depletion of microbiota by antibiotics ameliorated IMQ-treated psoriasis in mice, suggesting the role of microbiota in IMQ-induced skin inflammation^22,28^. Furthermore, treatment with IMQ caused altered composition of gut microbiota in mice^29^. Moreover, mice treated with antibiotics showed higher abundance of the genus *Lactobacillus* in the intestine and on the skin^30^.

Interestingly, it is recognized that the skin neuroendocrine system acts by preserving and maintaining the skin structural and functional integrity^31^, and that the skin is a sensory organ endowed with neuroendocrine activities^32^. However, as far as we know, there are no reports showing altered composition of microbes in the intestine and on the skin of IMQ-treated mice.

The present study was undertaken to examine whether topical application of IMQ to skin could influence the composition of microbiota in the intestine and on the skin of adult mice. Using 16S rRNA sequencing, we analyzed the composition of microbiota in the intestine and on the skin of control and IMQ-treated mice. It is well known that short chain fatty acids (SCFAs), the main metabolites produced by microbiota in the gastrointestinal tract, play an important role in the metabolic functions in human and rodents^33–36^. Therefore, we measured levels of SCFAs (i.e., acetic acid, propionic acid, butyric acid, lactic acid, and succinic acid) in the fecal samples.

## Results

### Effects of IMQ on the psoriasis-like score, body weight changes, spleen weight, and pathology of back skin

**Figure 1:**
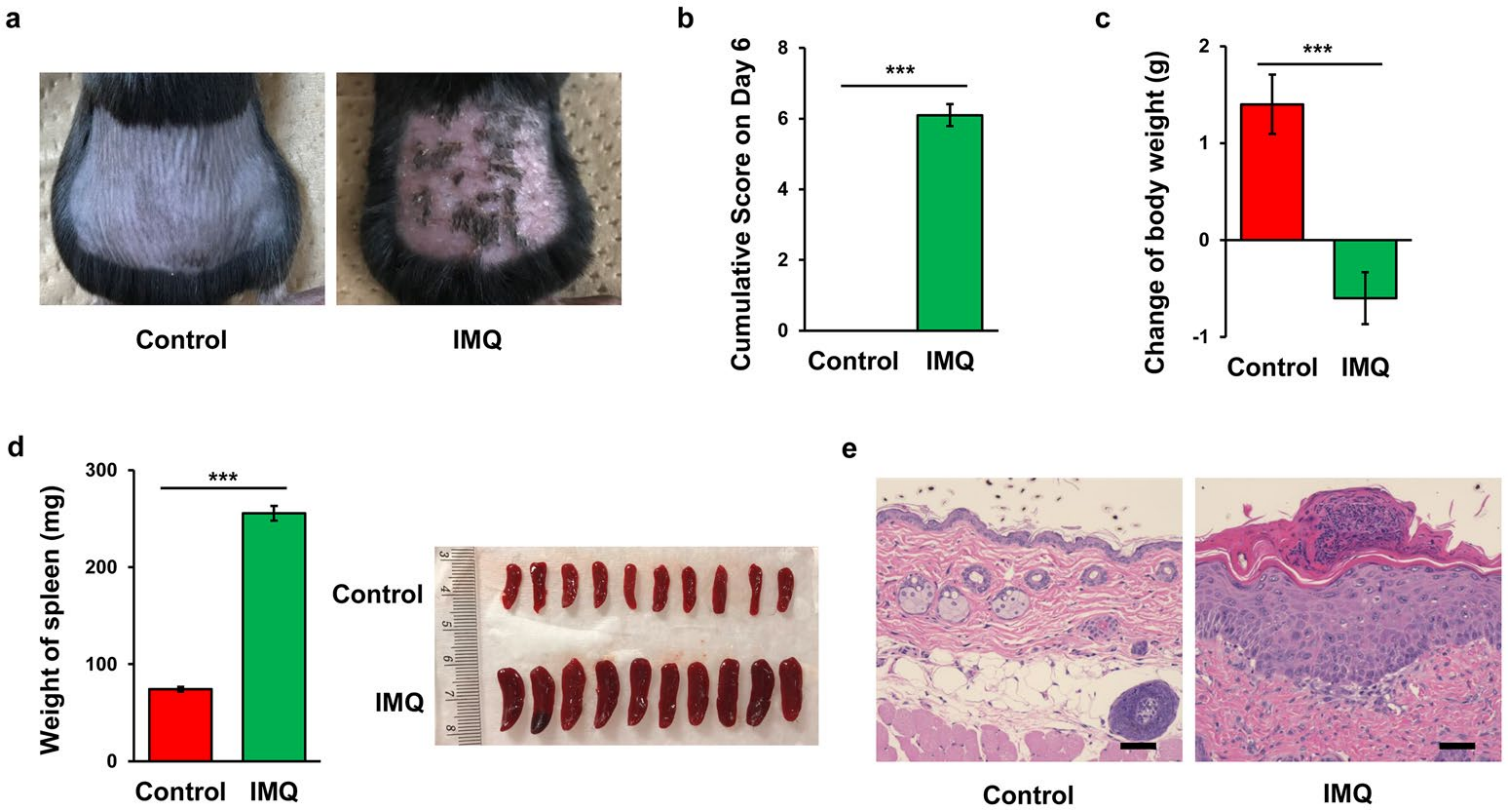
IMQ-induced psoriasis-like model. (**a**) Mice were treated topically with 5% IMQ cream or control cream on the shaved backs for five consecutive days. The representative photos of back skin from the two groups. (**b**) Cumulative score on day 6 of two groups (*P* < 0.001). (**c**) Change of body weight for the two groups (*P* < 0.001). (**d**) Weight of spleen (*P* < 0.001). The photos of spleen from the control and IMQ group. (**e**) HE staining of the back skin of the two groups. Scale bar = 50 μm. The values represent the mean ± S.E.M. (n = 10). ***P < 0.001.

IMQ-treated mice developed psoriasis-like dermatitis compared to control mice (Fig. 1a). Cumulative score of IMQ-treated mice was significantly higher than that of control mice (Fig. 1b). Furthermore, the body weight in the IMQ-treated mice was significantly lower than that of control mice (Fig. 1c). Moreover, the weight of spleen in the IMQ-treated mice was significantly higher than that of control mice (Fig. 1d), consistent with previous reports^27,37–39^. Representative H&E staining in the back skin showed parakeratosis with microabscess, acanthosis and an inflammatory infiltrate of the upper dermis in the IMQ-treated mice (Fig. 1e).

### Composition of gut microbiota

**Figure 2:**
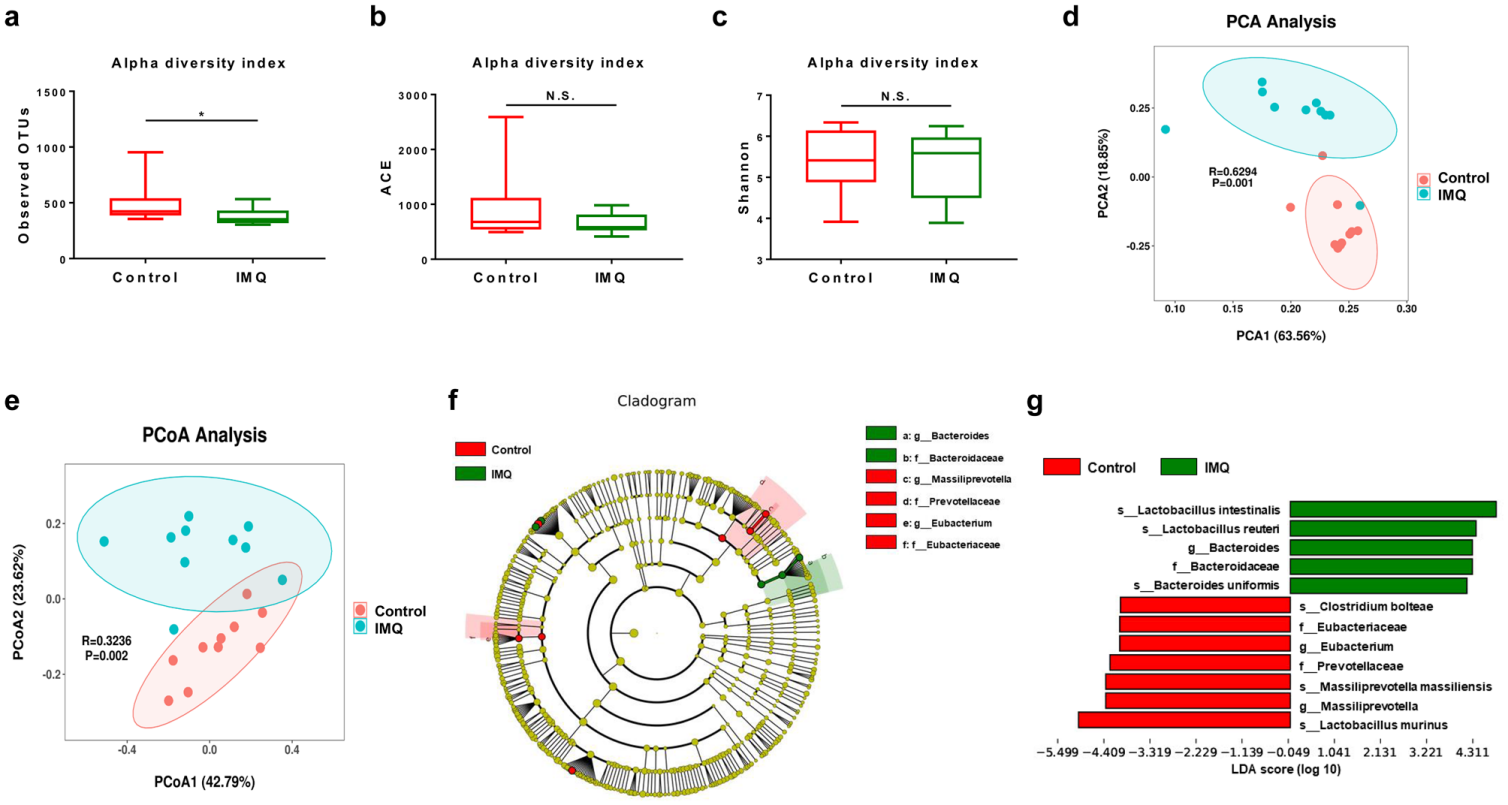
Effects of IMQ on the composition of gut microbiota in alpha-diversity and beta-diversity. (**a**) Alpha-diversity index of observed OTUs (Mann–Whitney U test, U = 21, *P* = 0.029). (**b**) Alpha-diversity index of ACE (Mann–Whitney U test, U = 21, *P* = 0.353). (**c**) Alpha-diversity index of Shannon (Mann–Whitney U test, U = 45, *P* = 0.739). (**d**) PCA of beta-diversity based on the OTU level (ANOSIM, R = 0.6294, *P* = 0.001). Each point represents a single sample which is color-coded according to group, and the two principal components (PC1 and PC2) explained 63.56% and 18.85%. (**e**) PCoA plot using weighted UniFrac distance (ANOSIM, R = 0.3236, *P* = 0.002). Each point represents a single sample and the two principal components (PCoA1 and PCoA2) explained 42.79% and 23.62%. (**f**) LEfSe cladogram (LDA score > 4.0, *P* < 0.05) indicated differentially abundant taxa between control group and IMQ group. Each circle represents the taxonomic categories from the species level as the outermost circle to phylum level as the innermost cycle. (**g**) Histograms revealed differentially abundant taxa with LDA score (log10) > 4.0 and *P* < 0.05 between the control group and IMQ group. The LDA scores of the control group was negative, while those of the IMQ group was positive. The values represent the mean ± S.E.M. (n = 10). *P < 0.05. N.S.: not significant.

Using alpha- and beta-diversity, we examined the composition of the gut microbiota in fecal samples. Mann–Whitney U-test showed a significant difference in the observed OTUs (Fig. 2a). In contrast, there were no changes for ACE and Shannon indices between the two groups (Fig. 2b,c). Beta diversity based on analysis of similarities (ANOSIM) was used to measure the degree of difference in bacterial community, including principal component analysis (PCA) of the OUT composition and principal coordinate analysis (PCoA) of weighted UniFrac distances. PCA demonstrated a significant difference in the microbiome (ANOSIM, R = 0.6294, *P* = 0.001) (Fig. 2d). In addition, PCoA of weighted UniFrac distances indicated a clear separation of IMQ group from control group (ANOSIM, R = 0.3236, *P* = 0.002) (Fig. 2e).

The changes of the abundant taxa were analyzed by the LEfSe algorithm. The color differences illustrated differences in the abundant taxa between the two groups. LEfSe analysis showed that IMQ group produced significant different effects on gut microbiota (Fig. 2f). Five mixed-level phylotypes, including the species *Lactobacillus intestinalis*, *Lactobacillus reuteri*, *Bacteroides uniformis*, the genus *Bacteroides*, and the family *Bacteroidaceae*, were identified as potential gut microbial markers for the IMQ group (Fig. 2g).

### Composition of the gut microbiota at the taxonomic level

At the phylum, there were no changes between the two groups (Figure S1). At the genus level, the abundance of *Bacteroides*, *Parabacteroides*, *Staphylococcus, Faecalimonas*, and *Alistipes* was significantly different between the two groups (Figure S2 and Table S1). At the species level, the abundance of *Lactobacillus murinus, Lactobacillus intestinalis, Lactobacillus reuteri, Lactobacillus taiwanensis, Bacteroides uniformis, Bacteroides acidifaciens, Bacteroides sartorii, Staphylococcus lentus, and Parabacteroides distasonis* was significantly different between the two groups (Figure S3 and Table S2).

### Composition of skin microbiota

**Figure 3:**
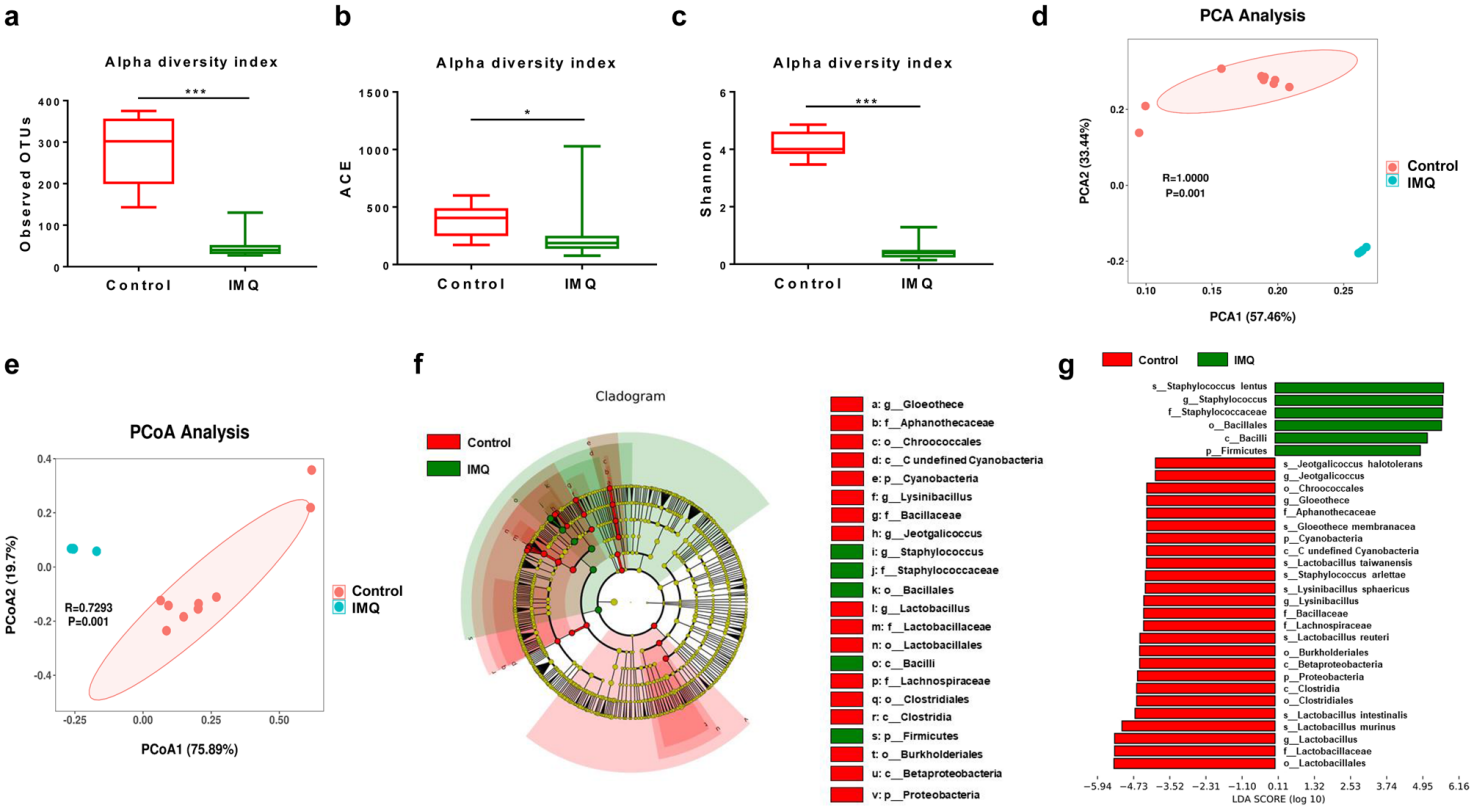
Effects of IMQ on the composition of skin microbiota in alpha-diversity and beta-diversity. (**a**) Alpha-diversity index of observed OTUs (Mann–Whitney U test, U = 0, *P* = 0.000). (**b**) Alpha-diversity index of ACE (Mann–Whitney U test, U = 20, *P* = 0.023). (**c**) Alpha-diversity index of Shannon (Mann–Whitney U test, U = 0, *P* = 0.000). (**d**) PCA of beta-diversity based on the OTU level (ANOSIM, R = 1.000, *P* = 0.001). Each point represents a single sample color-coded according to group, and the two principal components (PC1 and PC2) explained 57.46% and 33.44%. (**e**) PCoA plot using upon weighted UniFrac distance (ANOSIM, R = 0.7293, *P* = 0.001). Each point represents a single sample and the two principal components (PCoA1 and PCoA2) explained 75.89% and 19.7%. (**f**) LEfSe cladogram (LDA score > 4.0, *P* < 0.05) indicated differentially abundant taxa between control group and IMQ group. Each circle represents the taxonomic categories from the species level as the outermost circle to phylum level as the innermost cycle. (**g**) Histograms revealed differentially abundant taxa with LDA score (log10) > 4.0 and *P* < 0.05 between the control group and IMQ group. The LDA scores of the control group was negative, while those of the IMQ group was positive. The values represent the mean ± S.E.M. (n = 10). *P < 0.05; ***P < 0.001.

The composition of the microbiota on the back skin was analyzed. Mann–Whitney U-test showed significant differences in the observed OTUs, ACE, and Shannon (Fig. 3a–c). Beta-diversity analysis using PCA demonstrated significant differences between IMQ group and control group (ANOSIM, R = 1.000, *P* = 0.001) (Fig. 3d). Furthermore, significant separation was observed between the two groups on PCoA of weighted UniFrac distances (ANOSIM, R = 0.7293, *P* = 0.001) (Fig. 3e).

LEfSe difference analysis showed that IMQ group produced significant differential effects on skin microbiota (Fig. 3f). Six mixed-level phylotypes, including the species *Staphylococcus lentus*, the genus *Staphylococcus*, the family *Staphylcoccaceae*, the order *Bacillales*, the class *Bacilli*, and the phylum *Firmicutes* were identified as potential skin microbial markers for the IMQ group (Fig. 3g).

### Composition of the skin microbiota at the taxonomic level

At the phylum level, the abundance of *Firmicutes, Proteobacteria, Cyanobacteria, Actinobacteria,* and *Bacteroidetes* was significantly different between the two groups (Figure S4 and Table S3). At the genus level, the most abundant bacteria on the skin of IMQ-treated mice was *Staphylococcus* (the mean = 98.5%) whereas the level of *Staphylococcus* on the skin of control mice was low (the mean = 16.2%) (Figure S5). Furthermore, the abundance of sixteen bacteria was significantly different between the two groups (Figure S5 and Table S4). At the species level, the most abundant bacteria on the skin of IMQ-treated mice was *Staphylococcus lentus* (the mean is 93.5%) whereas the level of *Staphylococcus lentus* on the skin of control mice was very low (the mean is 7.14%) (Figure S6). Furthermore, the abundance of fourteen bacteria were significantly different between the two groups (Figure S6 and Table S5).

### Correlations between the skin microbiota and the gut microbiota

**Figure 4:**
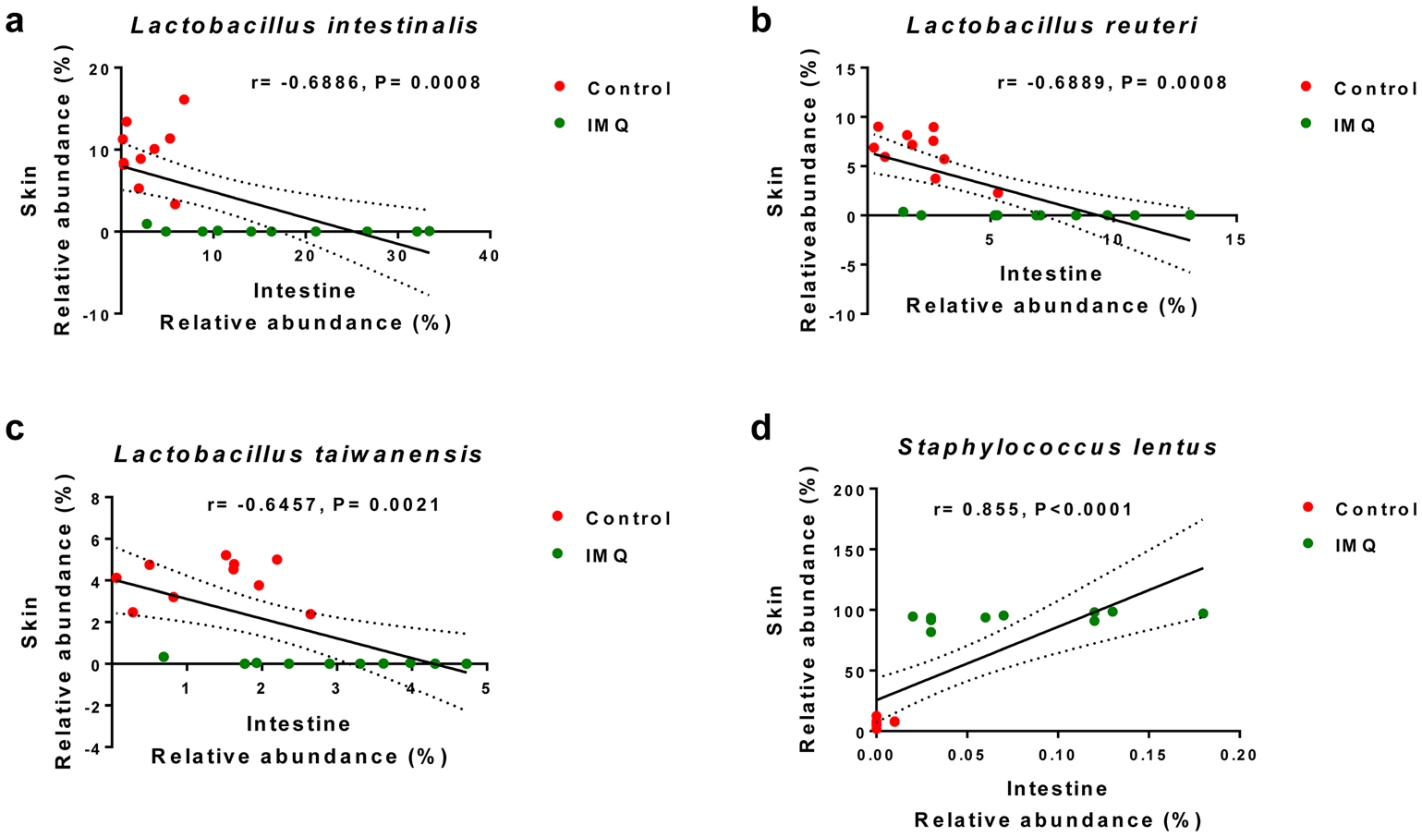
The correlations between bacterial relative abundance in the intestine and skin. (**a**) There was a significant negative correlation (r = − 0.6886, *P* = 0.0008) of the relative abundance of the species *Lactobacillus intestinalis* between the intestine and the skin. (**b**) There was a significant negative correlation (r = − 0.6889, *P* = 0.0008) of the relative abundance of the species *Lactobacillus reuteri* between the intestine and the skin. (**c**) There was a significant negative correlation (r = − 0.6457, *P* = 0.0021) of the relative abundance of the species *Lactobacillus taiwanensis* between the intestine and the skin. (**d**) There was a significant positive correlation (r = 0.855, *P* < 0.0001) of the relative abundance of the species *Staphylococcus lentus* between the intestine and the skin. The values represent the mean ± S.E.M. (n = 10).

At the species level, we found several microbes which were significantly altered in the intestine and on the skin between the two groups (Tables S2 and S5). Interestingly, there were negative correlations for *Lactobacillus intestinalis*, *Lactobacillus reuteri*, and *Lactobacillus taiwanensis* between the intestine and the skin (Fig. 4a–c). In contrast, there was a positive correlation for *Staphylococcus lentus* between the intestine and the skin (Fig. 4d).

### SCFAs in fecal samples and their correlations with the relative bacterial abundance

The levels of succinic acid and lactic acid in the IMQ group were significantly higher than the control groups (Table 1). In contrast, there were no changes for acetic acid, propionic acid, and n-butyric acid between the two groups (Table 1).

**Table 1:** Levels of short-chain fatty acids (SCFAs) in the fecal samples.

SCFAs (mg/g)	Control	IMQ	Student's t-test
**Succinic acid**	**0.146 ± 0.020**	**0.335 ± 0.065**	**df = 18, t = **− **2.767, *P* = 0.013**
**Lactic acid**	**1.141 ± 0.107**	**1.835 ± 0.289**	**df = 18, t = **− **2.252, *P* = 0.037**
Acetic acid	1.877 ± 0.341	1.750 ± 0.130	df = 18, t = 0.346, *P* = 0.733
Propionic acid	0.299 ± 0.033	0.372 ± 0.027	df = 18, t = − 1.704, *P* = 0.106
n-butyric acid	0.865 ± 0.225	0.907 ± 0.209	df = 18, t = − 0.137, *P* = 0.893

The values are the mean ± S.E.M. (n = 10).

Bold was statistically significant.

**Figure 5:**
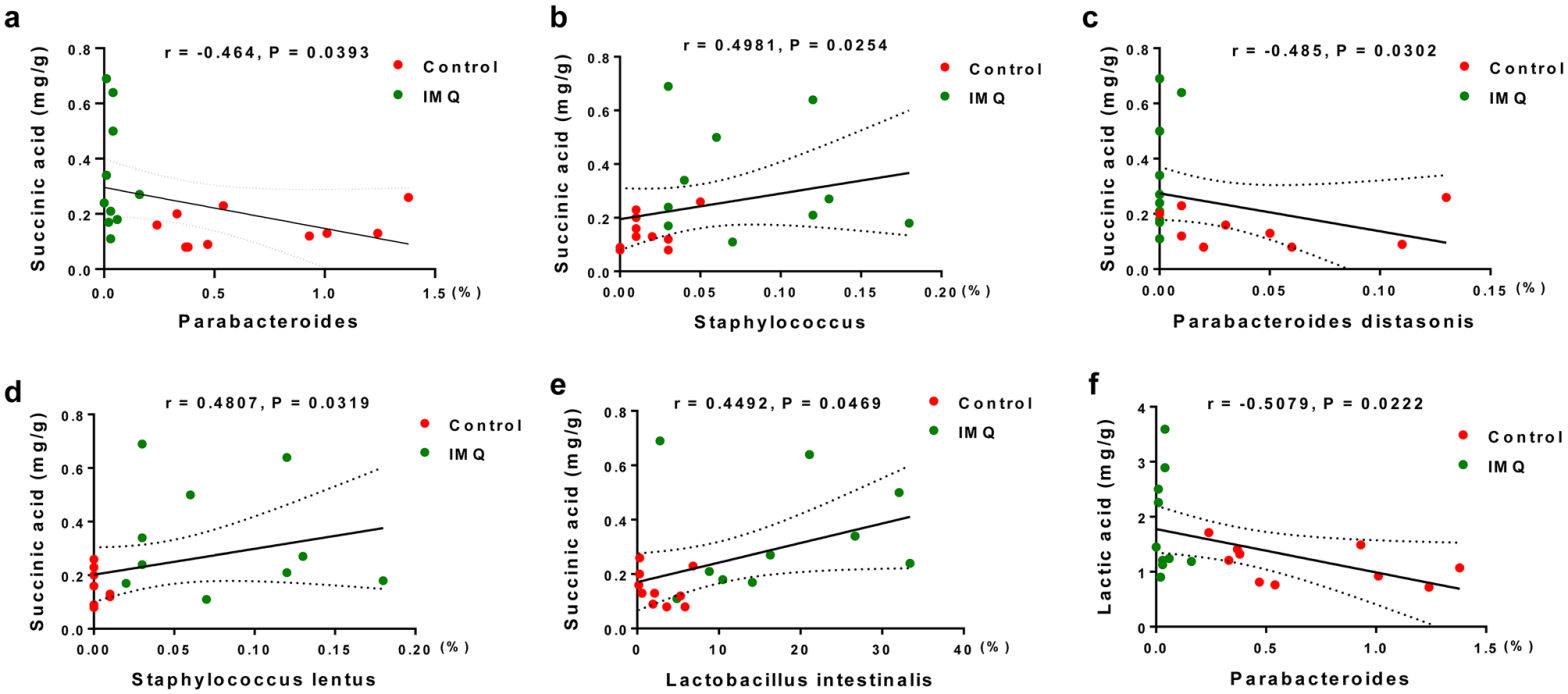
The correlations between bacterial relative abundance and SCFAs. (**a**) A significant negative correlation (r = − 0.464, *P* = 0.0393) between the relative abundance of the genus *Parabacteroides* and succinic acid was shown in the control group and IMQ group. (**b**) A significant positive correlation (r = 0.4981, *P* = 0.0254) between the relative abundance of the genus *Staphylococcus* and succinic acid was shown in the two groups. (**c**) A significant negative correlation (r = − 0.485, *P* = 0.0302) between the relative abundance of the species *Parabacteroides distasonis* and succinic acid was shown in the two groups. (**d**) A significant positive correlation (r = 0.4807, *P* = 0.0319) between the relative abundance of the species *Staphylococcus lentus* and succinic acid in the two groups. (**e**) A significant positive correlation (r = 0.4492, *P* = 0.0469) between the relative abundance of the species *Lactobacillus intestinalis* and succinic acid in the two groups. (**f**) There was a significant negative correlation (r = − 0.5079, *P* = 0.0222) between the relative abundance of the genus *Parabacteroides* and lactic acid in the two groups. The values represent the mean ± S.E.M. (n = 10).

Next, we examined the possible correlations between the relative abundance of microbes and SCFA levels in fecal samples. The succinic acid was significantly correlated with the relative abundance of the genus *Parabacteroides* (r = − 0.464, *P* = 0.0393) (Fig. 5a), the genus *Staphylococcus* (r = 0.4981, *P* = 0.0254) (Fig. 5b), the species *Parabacteroides distasonis* (r = − 0.485, *P* = 0.0302) (Fig. 5c), the species *Staphylococcus lentus* (r = 0.4807, *P* = 0.0319) (Fig. 5d) and the species *Lactobacillus intestinalis* (r = 0.4492, *P* = 0.0469) (Fig. 5e) in the two groups. A significant negative correlation between the relative abundance of the genus *Parabacteroides* (r = − 0.5079, *P* = 0.0222) and lactic acid was observed in two groups (Fig. 5f).

### Predictive functional metagenomes

**Figure 6:**
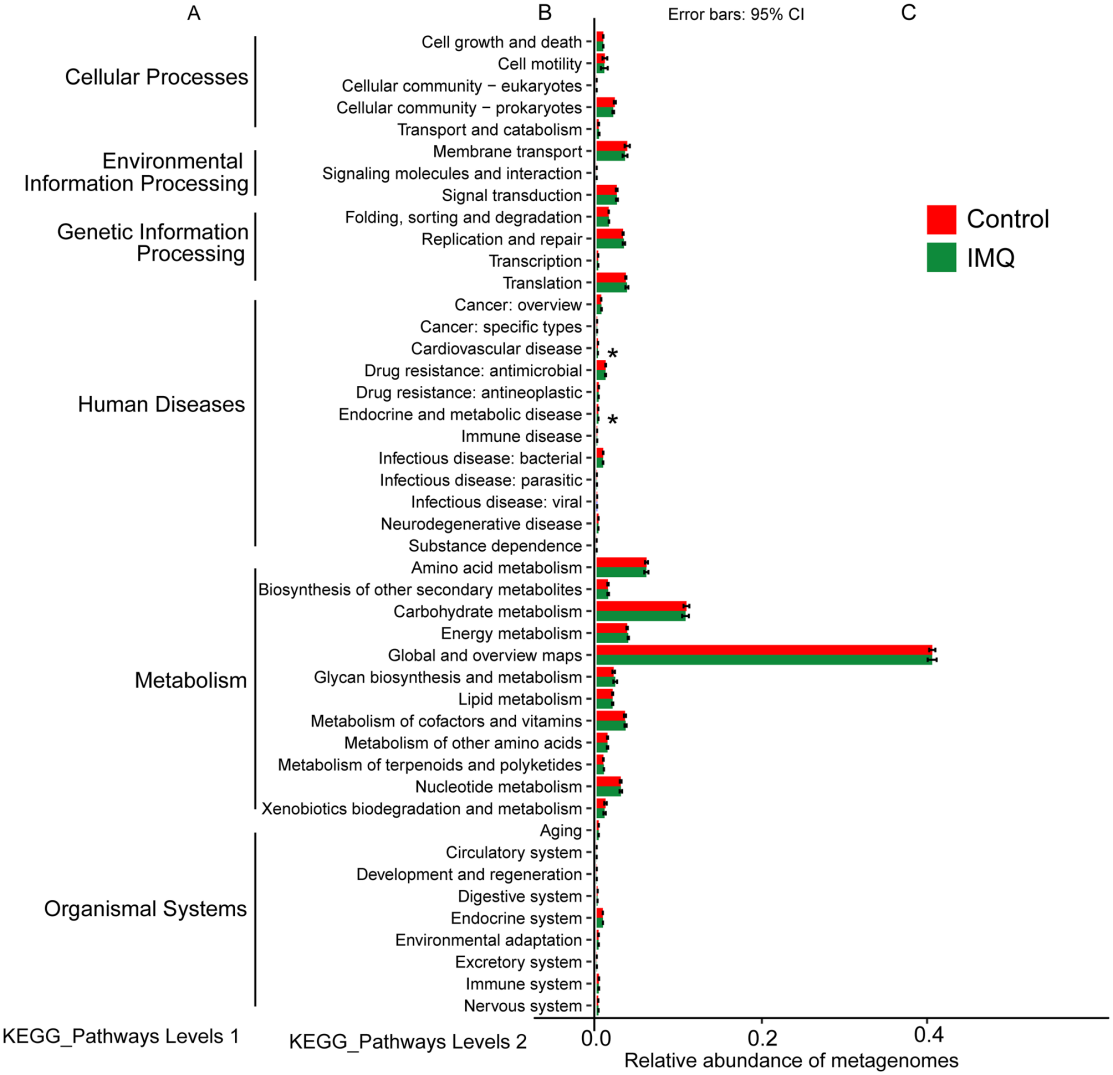
Relative abundance of KEGG pathways of functional categories in the gut microbiota. Functional predictions of the gut microbiota between the control group and IMQ group. Significant differences of KEGG pathways at level 2 were detected using STAMP software based on the KEGG pathway database (www.kegg.jp/kegg1.html). *P < 0.05.

**Figure 7:**
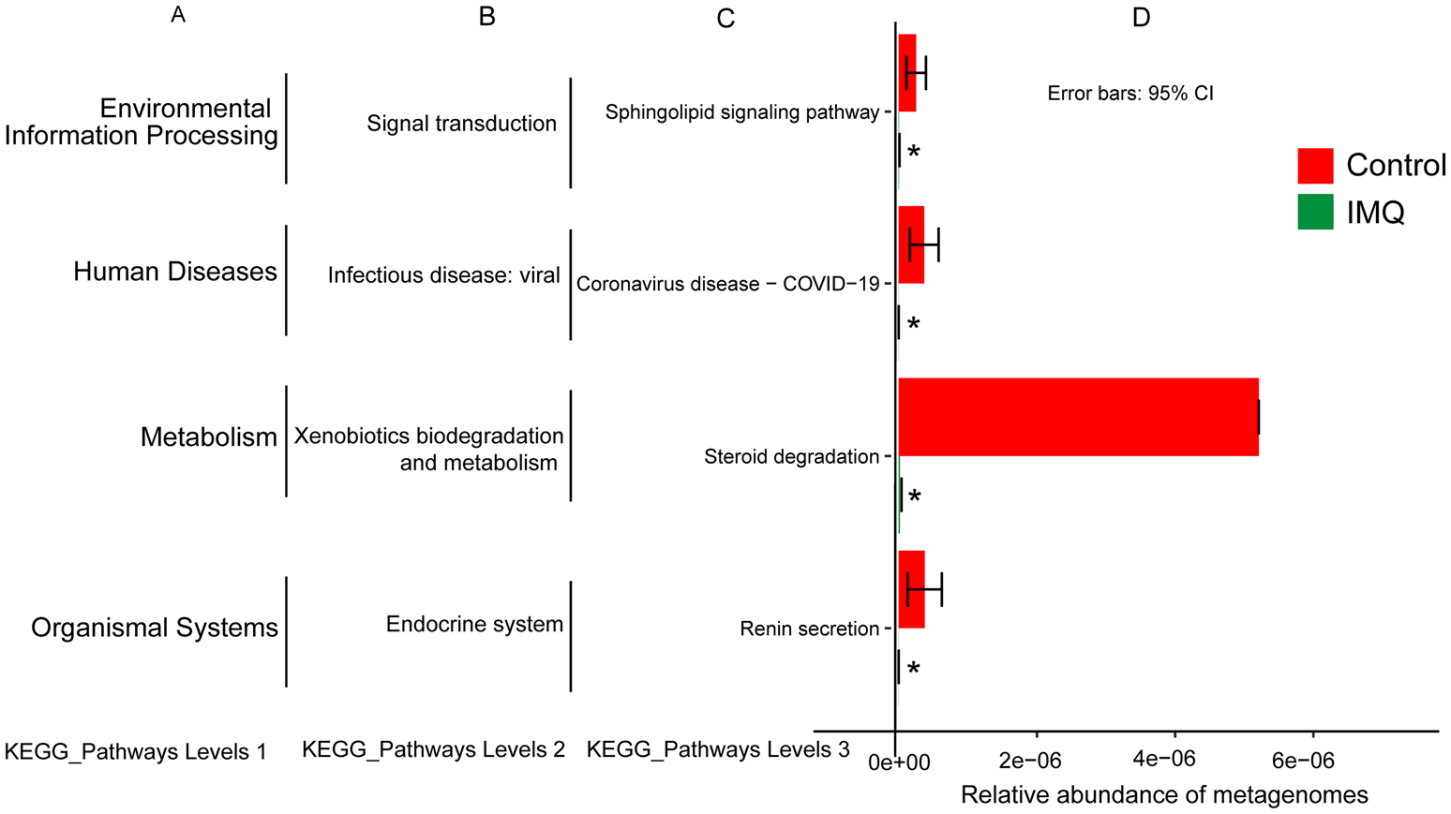
Relative abundance of KEGG pathways of functional categories in the skin microbiota. Functional predictions of the skin microbiota between the control group and IMQ group. Significant differences of KEGG pathways at level 3 were detected using STAMP software based on the KEGG pathway database (www.kegg.jp/kegg1.html). *P < 0.05.

In gut microbiota, two pathways on KEGG level 2, including cardiovascular disease, and endocrine and metabolic disease were significantly different between the two groups (Fig. 6). In skin microbiota, four pathways on KEGG level 3, including sphingolipid signaling pathway, coronavirus disease (COVID-19), steroid degradation, and renin secretion were significantly different between the two groups (Fig. 7).

## Discussion

The major findings of this study were as follows. First, topical application of IMQ to skin caused psoriasis-like phenotypes in mice. Second, IMQ caused significant alterations in the alpha- and beta-diversity of the microbiota in the intestine and on the skin. The LEfSe algorithm of gut microbiota identified the species *Lactobacillus intestinalis*, *Lactobacillus reuteri*, and *Bacteroides uniformis* as potential gut microbial markers for the IMQ group. Furthermore, the LEfSe algorithm of skin microbiota identified the species *Staphylococcus lentus* as potential skin microbial marker for the IMQ group. Interestingly, correlations for several microbes between the intestine and the skin were observed, suggesting a role of skin–gut–microbiota in IMQ-treated mice. Third, levels of succinic acid and lactic acid in feces increased in the IMQ group compared to control group. Interestingly, we found that the levels of succinic acid (or lactic acid) were correlated with the relative abundance of several microbes in the fecal samples. Finally, the predictive functional analysis of the microbiota of gut and skin showed that IMQ caused alterations in several KEGG pathways. Taken all together, the present data show that topical treatment with IMQ alters the composition of the microbiota in the intestine and on the skin of host.

At the species level, the three *Lactobacillus* microbes such as *Lactobacillus intestinalis*, *Lactobacillus reuteri*, and *Lactobacillus taiwanensis* were significantly higher in the intestine of IMQ-treated mice compared to control mice. A recent study showed that *Lactobacillus intestinalis* and *Lactobacillus reuteri* may be responsible for the depression-like behavior in mice after transplantation of “depression-related microbes”^40^. The increased abundance of *Lactobacillus intestinalis, Lactobacillus reuteri* and *Lactobacillus taiwanensis* by IMQ treatment may contribute to the increased levels of lactic acid in the IMQ-treated mice since *Lactobacilli* ferment lactose into lactic acid^41^. Collectively, it is likely that increased abundance of *Lactobacillus* bacteria may contribute to increased levels of lactic acid in the host gut although further detailed study is needed. Considering the beneficial actions of *Lactobacillus reuteri* on the host immune system^41,42^, it seems that IMQ-induced increases in the abundance of bacteria might reflect a compensatory response in the host. There are many species of *Lactobacillus* which may have beneficial and harmful effects in the host^43^. Nonetheless, future studies are needed to investigate the mechanisms underpinning increases of these *Lactobacillus* bacteria in the intestine of IMQ-treated mice.

Furthermore, we found significant differences for *Bacteroides uniformis, Bacteroides acidifaciens, Bacteroides sartorii, Staphylococcus lentus* and *Parabacteroides distasonis* in the intestine between the two groups. As far as we know, there are no reports showing alterations in these three *Bacteroides* bacteria in IMQ-treated mice and patients with psoriasis. Pretreatment with the antibiotic metronidazole increased the abundance of *Parabacteroides distasonis* in the intestine of IMQ-treated mice^30^. In contrast, *Parabacteroides distasonis* were significantly decreased in the patients with psoriasis^44^.

At the species level, we found many skin microbes which altered in the IMQ-treated mice (Table S5). Importantly, the most abundant microbe on the skin from IMQ-treated mice was *Staphylococcus lentus*, and the abundance of *Staphylococcus lentus* in the IMQ-treated mice were significantly higher than control mice (Table S5). *Staphylococcus lentus* are commensal bacterium colonizing the skin of animals and has been associated with infections in animals^45^. It seems that high abundance of *Staphylococcus lentus* may play a role in IMQ-induced psoriasis-like symptoms in mice. However, the precise mechanisms underlying high abundance of *Staphylococcus lentus* on the skin of IMQ-treated mice are currently unclear. Further study is needed to examine the role of *Staphylococcus lentus* in psoriasis-like symptom of IMQ-treated mice.

In this study, we found several microbes which altered in the both intestine and skin. Interestingly, we found significant correlations for *Lactobacillus intestinalis*, *Lactobacillus reuteri*, *Lactobacillus taiwanensis,* and *Staphylococcus lentus* between the intestine and the skin. To the best of our knowledge, this is the first report showing the correlations for microbes in both the intestine and the skin, supporting the skin–gut microbiota axis^46,47^. From the current data, it is unclear whether changes in gut microbiota can affect skin microbiota or these two phenomena are independent. In this study, we found that topical treatment with IMQ caused increased volume of spleen through systemic inflammation, resulting in abnormal changes in the microbiota composition in the intestine and on the skin of mice. Although the precise mechanisms underlying the association between the skin and the intestine remain unclear, the current data strongly suggest a role of skin–gut microbiota axis in IMQ-treated mice. Therefore, it is of great interest to investigate whether the composition of microbiota in the intestine and on the skin from patients with psoriasis is altered compared to healthy control subjects.

Succinic acid is produced in large amounts during bacterial fermentation of dietary fiber, and it is considered as a key intermediate in the synthesis of propionic acid^48,49^. Interestingly, germ-free mice have little or no detectable levels of succinic acid in feces compared to conventional mice, indicating that gut microbiota are the predominant source for succinic acid. Higher levels of succinic acid may be related with high abundance of *Bacteroides* in fecal samples since succinic acid is produced by primary fermenters such as *Bacteroides*^49^. It is also shown that elevated levels of succinic acid in the feces are associated with intestinal inflammation^48,49^. Collectively, higher levels of succinic acid in the feces of IMQ-treated mice might be associated with intestinal inflammation although further study is needed.

To understand the role of altered composition of microbiota in the intestine and skin of IMQ-treated mice, we examined the predictable function of the microbiota. The current data show that IMQ treatment may contribute to the altered metabolism (i.e., cardiovascular disease, and endocrine and metabolic disease) induced by gut microbiota. In addition, the current data show that IMQ treatment could produce the altered metabolism (i.e., sphingolipid signaling pathway, coronavirus disease—COVID-19, steroid degradation, and renin secretion) induced by skin microbiota. It is noteworthy that we could detect the altered metabolism of endocrine disease in IMQ-treated mice since the neuroendocrine system plays a role in the skin function^31,32^. Furthermore, it is likely that gut microbiota is more complex than skin microbiota since we detected many pathways for gut microbiota compared to skin microbiota. Taken together, it is likely that these KEGG pathways provide a new functional view for understanding the gut and skin microbiota that contribute to psoriasis-like symptoms. Finally, this study has a potential limitation. A future study using antibiotic cocktail is needed to ascertain the correlation between skin microbiota and gut microbiota in IMQ-treated mice.

In conclusion, this study shows that topical treatment with IMQ causes abnormal changes in the microbiota composition in the intestine and on the skin of adult mice, and that levels of succinic acid were associated with the relative abundance of several microbes. Furthermore, we found correlations for several microbes between the intestine and the skin, suggesting a role of skin–gut microbiota in psoriasis.

## Materials and methods

### Animals

Female C57BL/6 mice (9 weeks old, weighing 18–21 g, n = 20, Japan SLC Inc., Hamamatsu, Shizuoka, Japan) were used. Mice were housed (5 per cage) under a 12-h/12-h light/dark cycle (lights on between 07:00 and 19:00), with ad libitum access to food access to food (CE-2; CLEA Japan, Inc., Tokyo, Japan) and water. The experimental protocol was approved by Chiba University Institutional Animal Care and Use Committee (Permission number: 2-433)^50^. This study was carried out in strict accordance with the recommendations in the Guide for the Care and Use of Laboratory Animals of the National Institutes of Health, USA^50^. This study was also carried out in compliance with the ARRIVE guidelines. All efforts were made to minimize animal suffering.

### Treatment of IMQ and collection of samples

The shaved back skin of mice was treated with 62.5 mg of 5% IMQ cream (Beselna cream; Mochida Pharmaceutical Co., Tokyo, Japan) daily for 5 consecutive days. Control mice were treated similarly with 62.5 mg of white petrolatum (Maruishi Pharmaceutical Co., Osaka, Japan). Skin, fecal and spleen samples were collected on day 6. The clinical skin score was measured on day 1 and day 6. The degree of skin inflammation was scored by cumulative disease severity score, similar to the human Psoriasis Area and Severity Index, but not taking the area into account. Erythema, scaling, and thickening were scored independently from 0 to 4: 0, none; 1, slight; 2, moderate; 3, marked; 4, very marked. The single scores were summed, resulting in a theoretical maximal total score of 12^27^.

Fresh fecal samples of mice were collected from 7:30 to 8:30 on day 6 to exclude any circadian effects on the microbes. The fecal samples were placed into sterilized screw-cap microtubes immediately after defecation, and they were frozen in liquid-nitrogen immediately. The samples were stored at − 80 °C until use^51^.

Skin swabs from the shaved back skin of IMQ-treated mice or control mice were put into extraction tube containing a solution (0.15 M NaCl and 0.1% Tween20) and rotated for at least 20 times. After squeezing as much liquid as possible from the swab by pushing the swabs against the sides of the tubes, the tubes were stored at − 80 °C until analysis.

### Histology

Back skin samples from control and IMQ-treated groups were collected and fixed in 10% formalin (FUJIFILM Wako Pure Chemical Corp.). Staining with hematoxylin and eosin (HE) was performed at Biopathology Institute Co., Ltd (Kunisaki, Oita, Japan). Back skin samples were embedded in paraffin, and sections of 3 μm were prepared and subjected to HE staining. Representative images of two groups were obtained using a Keyence BZ-9000 Generation II microscope (Osaka, Japan).

### 16S rRNA analysis

The DNA extractions from the fecal and skin samples and 16S rRNA sequencing analyses were performed by MyMetagenome Co., Ltd. (Tokyo, Japan), as reported previously^40,50–52^. DNA extraction from mouse samples and purification were performed according to the method of the previous report^53,54^. The 16S rRNA analysis of samples was performed as previously reported^53,54^. Briefly, PCR was performed using 27Fmod 5′-AGRGTTTGATYMTGGCTCAG-3′ and 338R 5′-TGCTGCCTCCCGTAGGAGT-3′ to amplify the V1–V2 region of the bacterial 16S rRNA gene. The 16S amplicons were then sequenced using MiSeq according to the Illumina protocol. Taxonomic assignment of OTUs was made by similarity searches against the Ribosomal Database Project and the National Center for Biotechnology Information genome database using the GLSEARCH program.

Alpha diversity was used to analyze the species diversity, composed of richness and evenness, within a sample through three indices including the observed OTU, ACE, and Shannon indices^55^. Beta diversity was used to measure differences of species diversity among samples, including PCA and PCoA with ANOSIM. Differences in bacterial taxa between groups at the species or higher level (depending on the taxon annotation) were calculated based on linear discriminant analysis (LDA) effect size (LEfSe) using LEfSe software (LDA score > 4.0, *P* < 0.05)(https://www.omicstudio.cn/tool/)^56^.

### Prediction of functional profiles of gut microbiota using PICRUSt

Using the 16S rRNA gene sequencing data and KEGG (Kyoto Encyclopedia of Genes and Genome) orthology (http://www.kegg.jp/kegg1.html)^57^, we performed PICRUSt (Phylogenetic Investigation of Communities by Reconstruction of Unobserved States) analysis and STAMP (Statistical Analysis of Metagenomic Profiles) software v2.1.3 (http://kiwi.cs.dal.ca/Software/STAMP) for the functional prediction of microbiota in the intestine and skin^55,58,59^.

### Measurement of short-chain fatty acid (SCFA) levels

Concentrations of SCFAs (i.e., acetic acid, propionic acid, butyric acid, lactic acid, succinic acid) in fecal samples were measured at TechnoSuruga Laboratory, Co., Ltd. (Shizuoka, Japan), as reported previously^50,51,55,60^. The data of SCFAs were shown as milligrams per gram of feces.

### Statistical analysis

Data are shown as the mean ± standard error of the mean (S.E.M.). Alpha-diversity of the gut and skin microbiota were analyzed using Mann–Whitney U-test. Analysis of beta-diversity of the gut and skin microbiota including PCA of OTU level and PCoA of weighted UniFrac distances were performed based on ANOSIM by R package vegan (2.5.4) (https://CRAN.R-project.org/package=vegan)^61^. Data for SCFA levels were analyzed using Student t-test. Correlations between SCFAs and the relative bacterial abundance were analyzed using Spearman’s correlation analysis. Correlations between the relative abundance of bacteria in the skin and intestine were also analyzed using Spearman’s correlation analysis. *P* < 0.05 was considered statistically significant.

## Supplementary Information

Supplementary Information.

## Data Availability

The data that support the findings of this study are available from the corresponding author upon reasonable request.
