# Supplementary material for: Abnormal﻿ composition of microbiota in the gut and skin of imiquimod-treated mice
Source: Sci Rep. 2021 May 28;11:11265. doi: 10.1038/s41598-021-90480-4 (PMC8163751; doi:10.1038/s41598-021-90480-4)
Supplement: Supplementary file 1 — Supplementary Information. [file 41598_2021_90480_MOESM1_ESM.docx]

**Supplemental Information**

**Abnormal composition of microbiota in the gut and skin of imiquimod-treated mice**

Hiroyo Shinno-Hashimoto^1,2^, Yaeko Hashimoto^2,3^, Yan Wei^2,4^, Lijia Chang^2^,

Yuko Fujita^2^, Tamaki Ishima^2^, Hiroyuki Matsue^1^, and Kenji Hashimoto^2^

^1^Department of Dermatology, Chiba University Graduate School of Medicine, Chiba 260-8670, Japan, ^2^Division of Clinical Neuroscience, Chiba University Center for Forensic Mental Health, Chiba 260-8670, Japan, ^3^Department of Respirology, Chiba University Graduate School of Medicine, Chiba 260-8670, Japan, and ^4^Key Laboratory of Medical Electrophysiology of Ministry of Education and Medical Electrophysiological Key Laboratory of Sichuan Province, Collaborative Innovation Center for Prevention and Treatment of Cardiovascular Disease, Institute of Cardiovascular Research, Southwest Medical University, Luzhou, 646000, Sichuan, China

**Correspondence:** Prof. Kenji Hashimoto, Division of Clinical Neuroscience, Chiba University Center for Forensic Mental Health, Chiba 260-8670, JAPAN

Tel: +81-43-226-2517; Fax: +81-43-226-2561 (e-mail: hashimoto@faculty.chiba-u.jp)


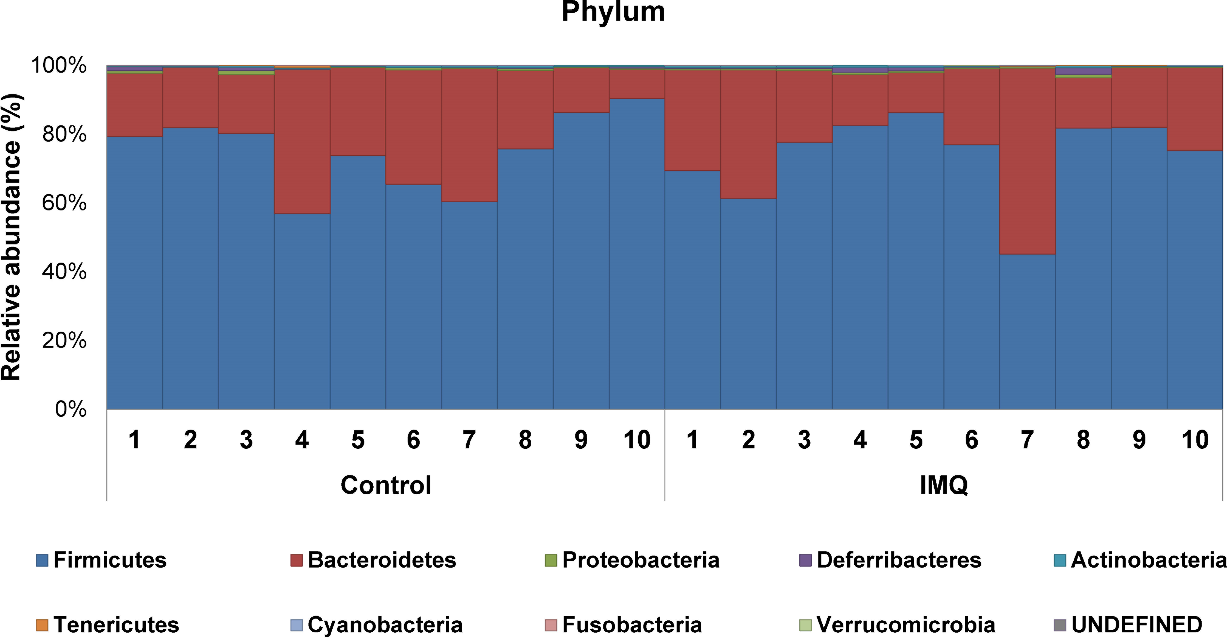


**Figure S1. The composition in the gut microbiota at the levels of phylum**

The relative abundances of gut microbiome at phylum level in fecal samples of the control and IMQ groups. There were no changes for microbes between the two groups.

**
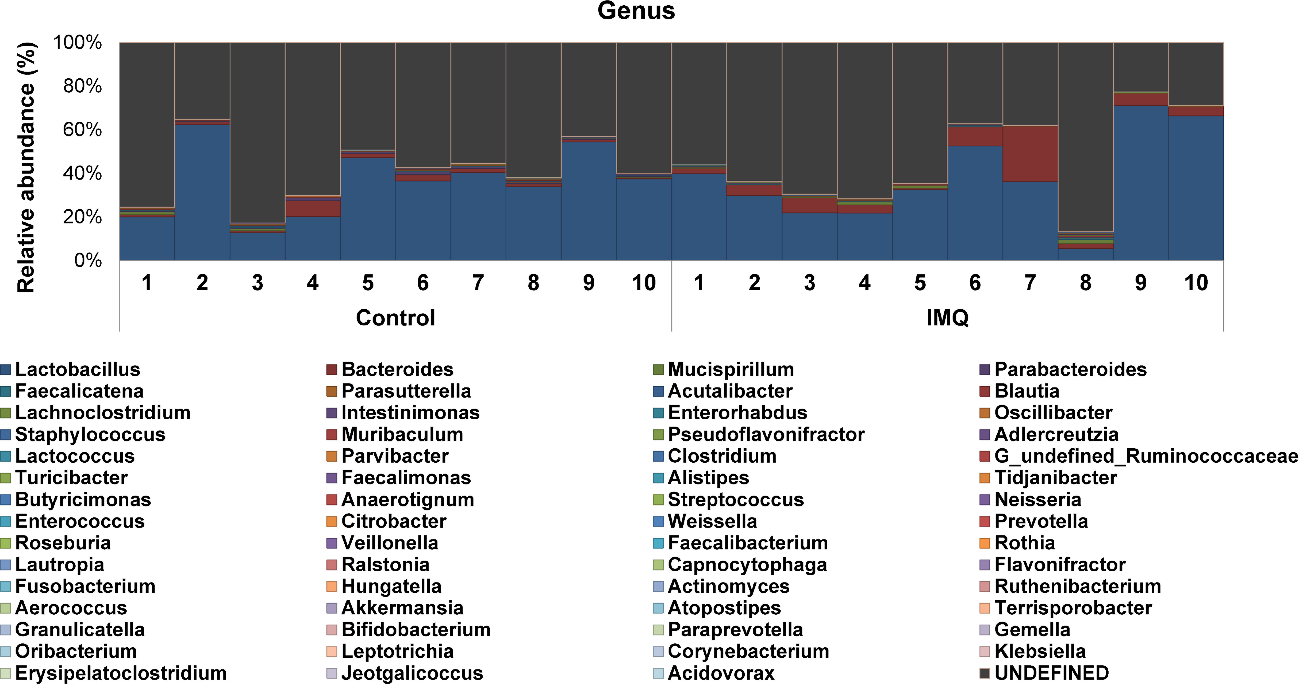
**

**Figure S2. The composition in the gut microbiota at the levels of genus**

The relative abundances of gut microbiome at genus level in fecal samples of the control and IMQ groups. There were significantly (P < 0.001) changes for *Parabacteroides*, *Staphylococcus*, and *Alistipes* between the two groups (**Table S1**).

**
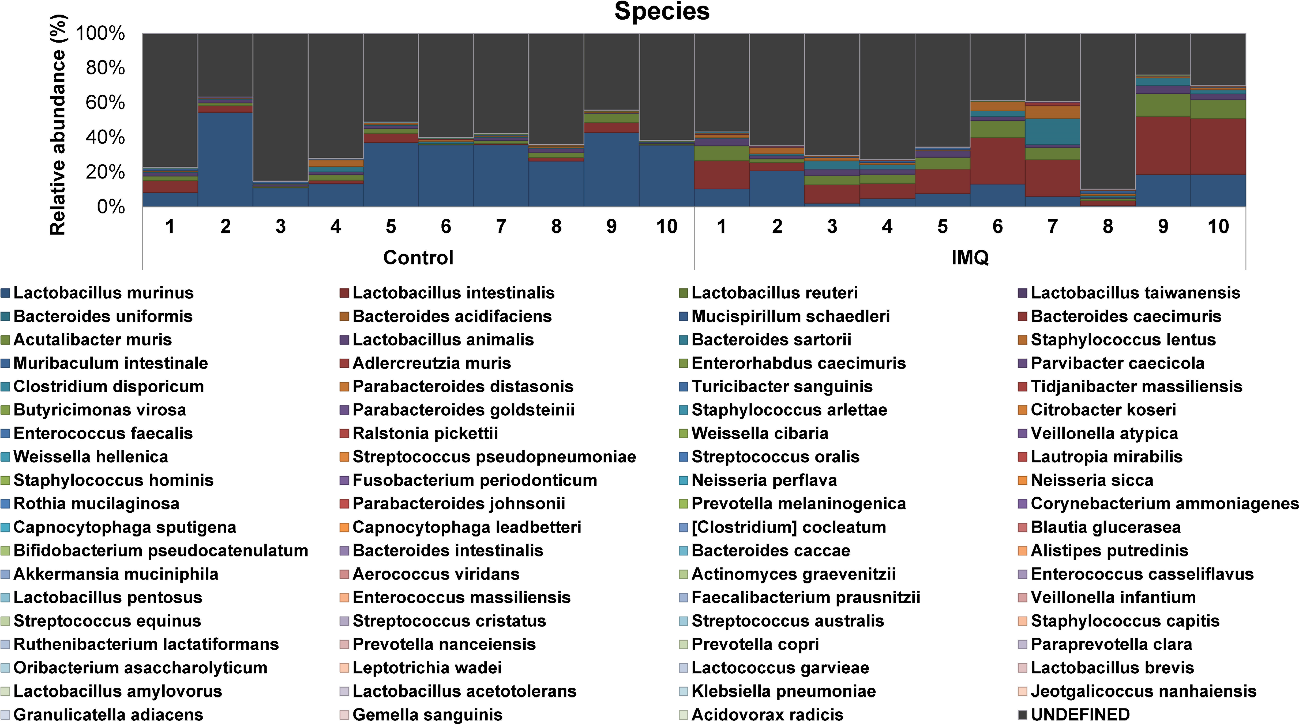
**

**Figure S3. The composition in the gut microbiota at the levels of species**

The relative abundances of gut microbiome at species level in fecal samples of the control and IMQ groups. There were significantly changes for several microbiome between the two groups (**Table S2**).

**
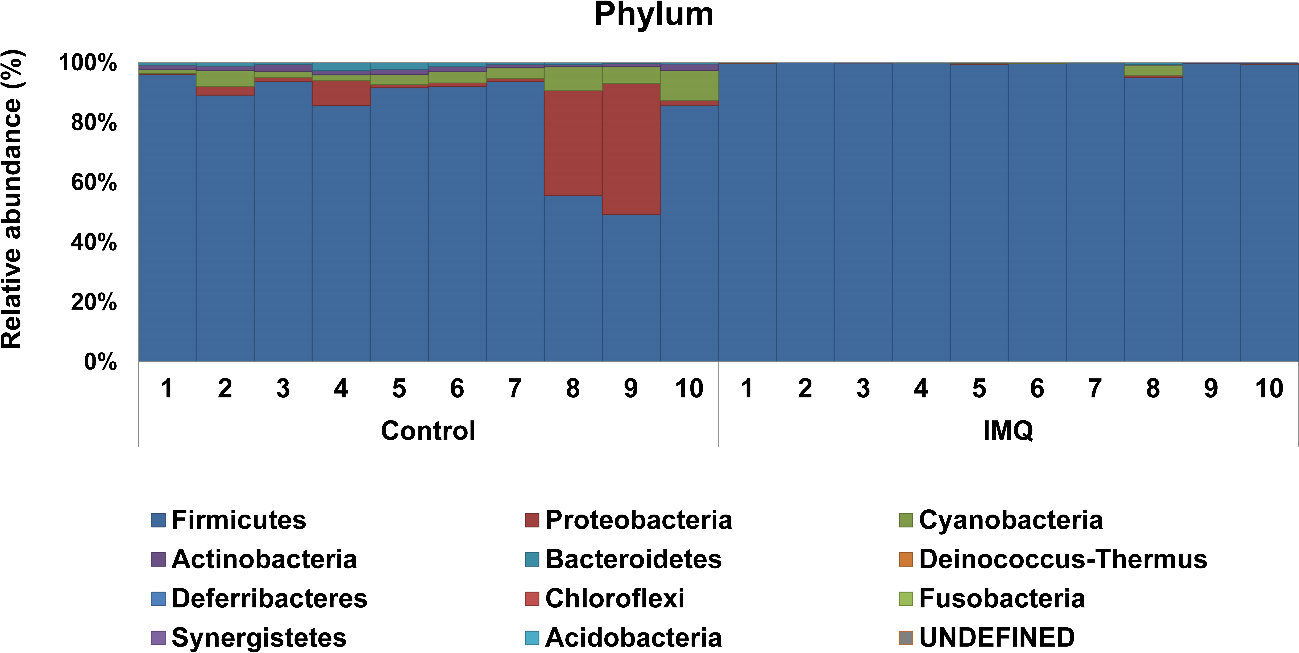
**

**Figure S4. The composition in the skin bacteria at the levels of phylum**

The relative abundances of skin microbiome at phylum level in skin samples of the control and IMQ groups. There were significantly (P < 0.001) changes for *Firmicutes*, *Proteobacteria, Cyanobacteria*, *Actinobacteria* and *Bacteroidetes* between the two groups (**Table S3**).

**
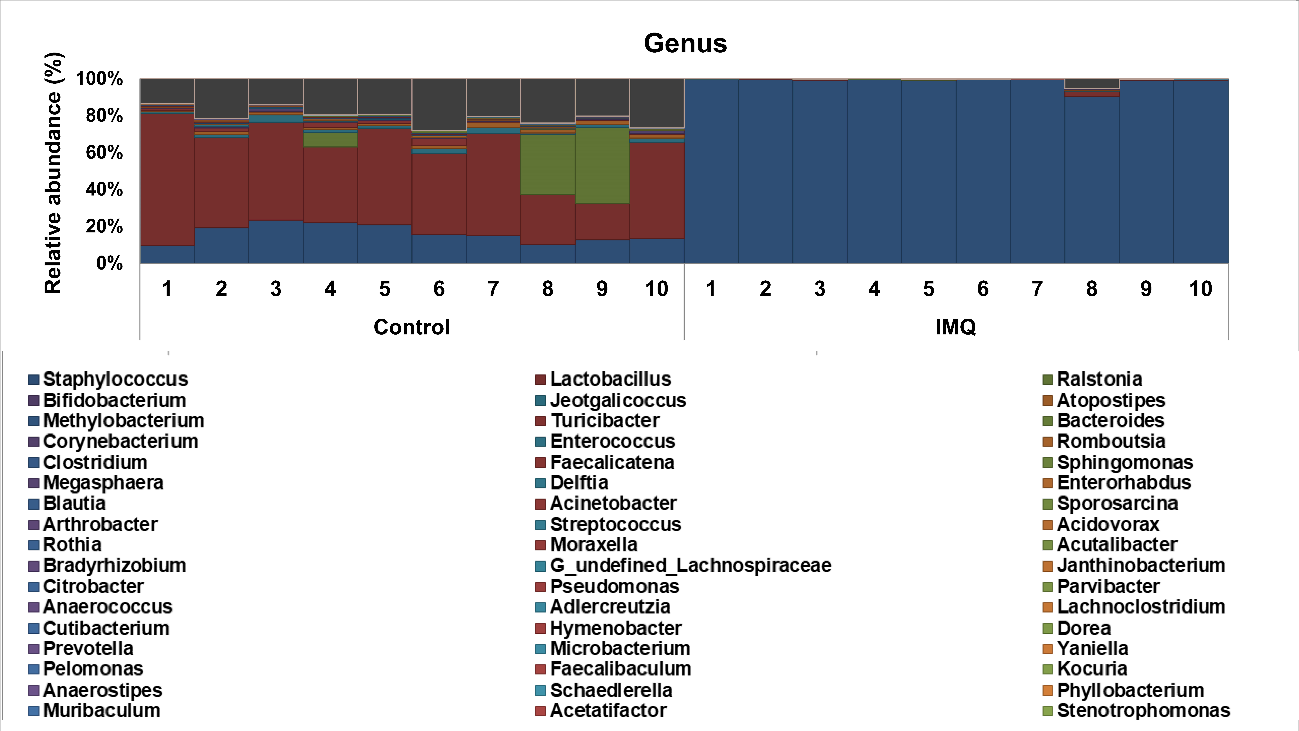
**

**
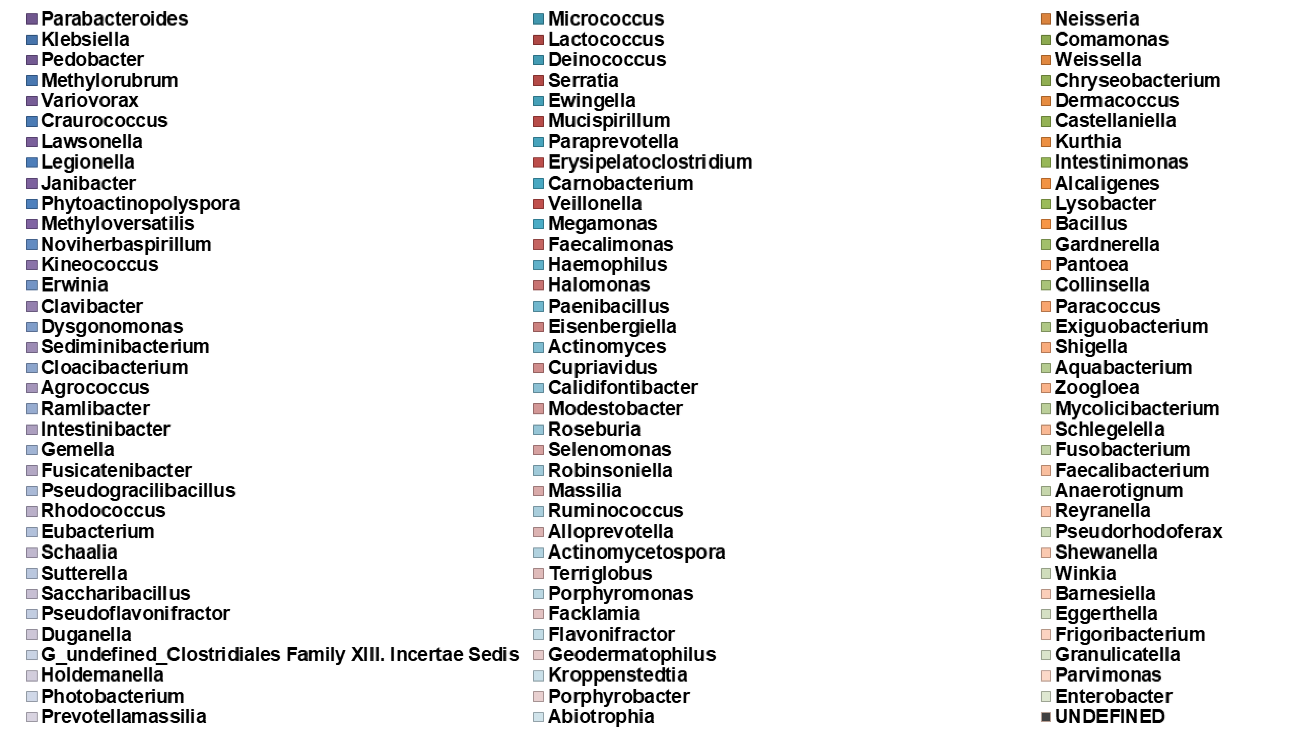
**

**Figure S5. The composition in the skin microbiota at the levels of genus**

The relative abundances of skin microbiome at genus level in skin samples of the control and IMQ groups. There were significantly changes for sixteen microbes between the two groups (**Table S4**).

**
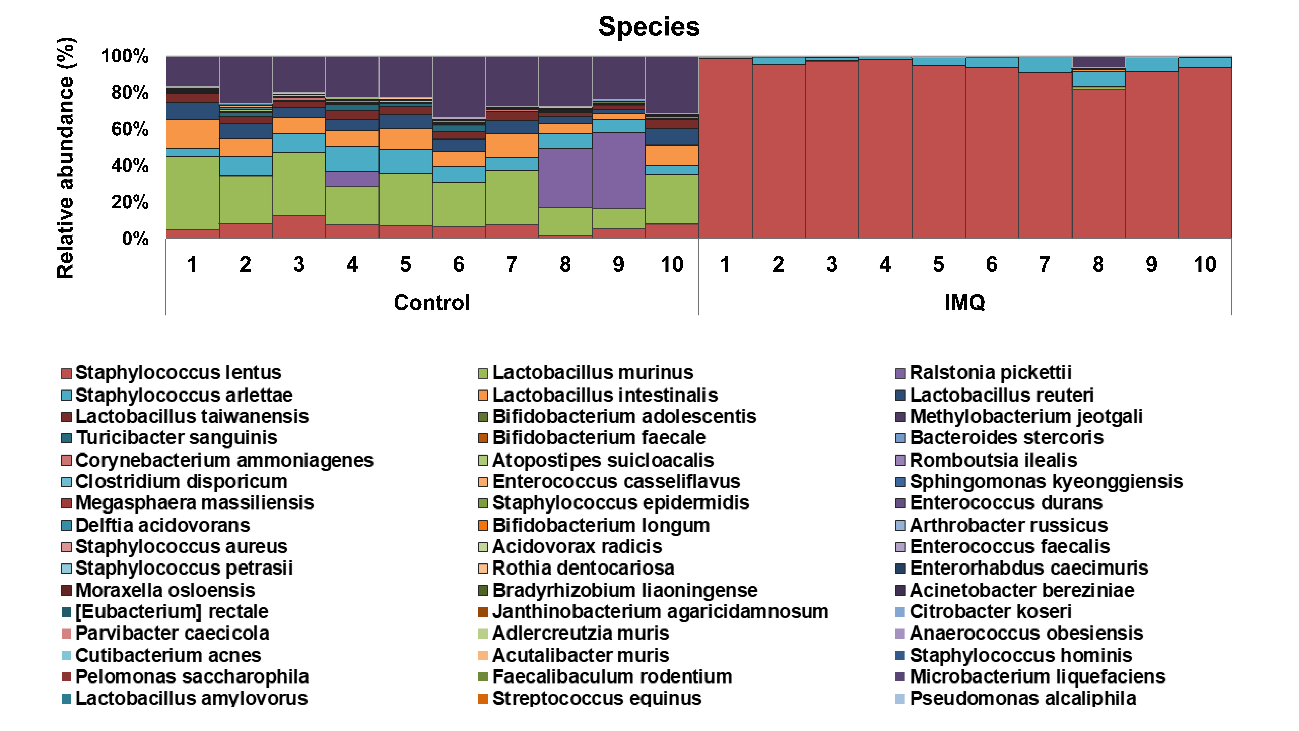
**

**
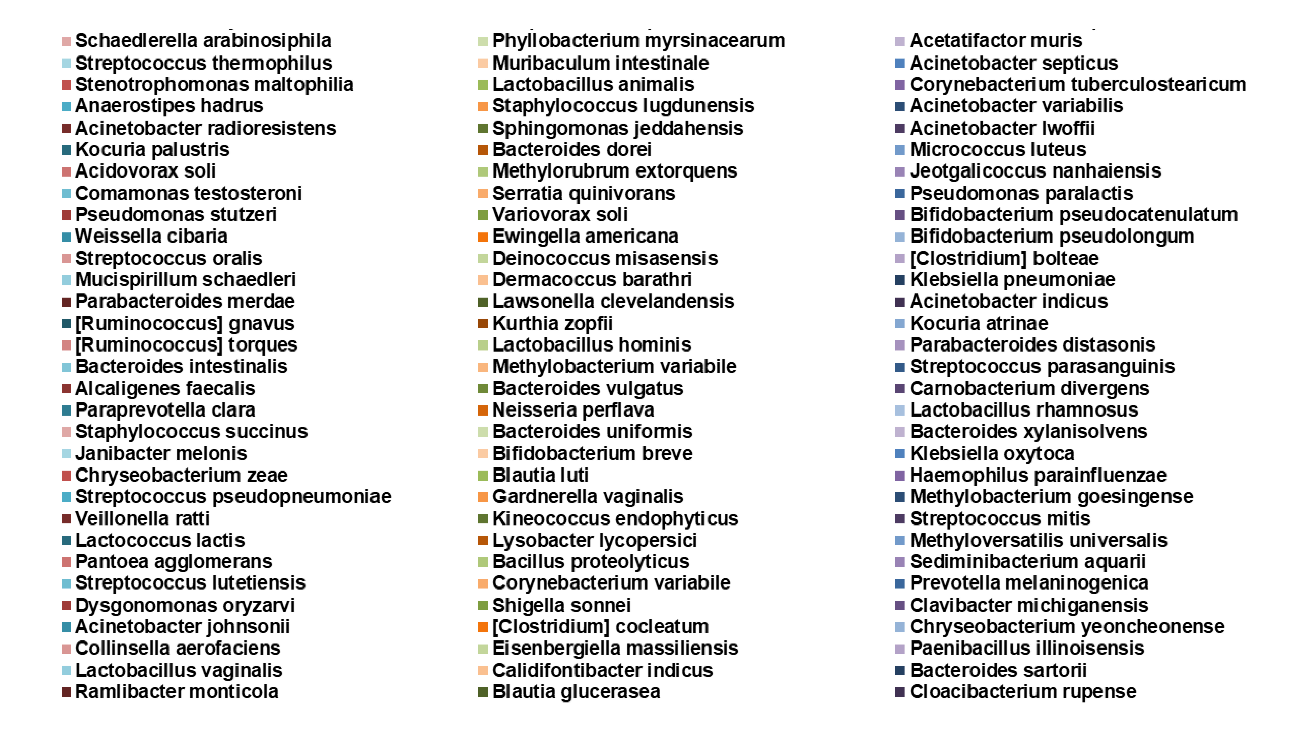
**

**
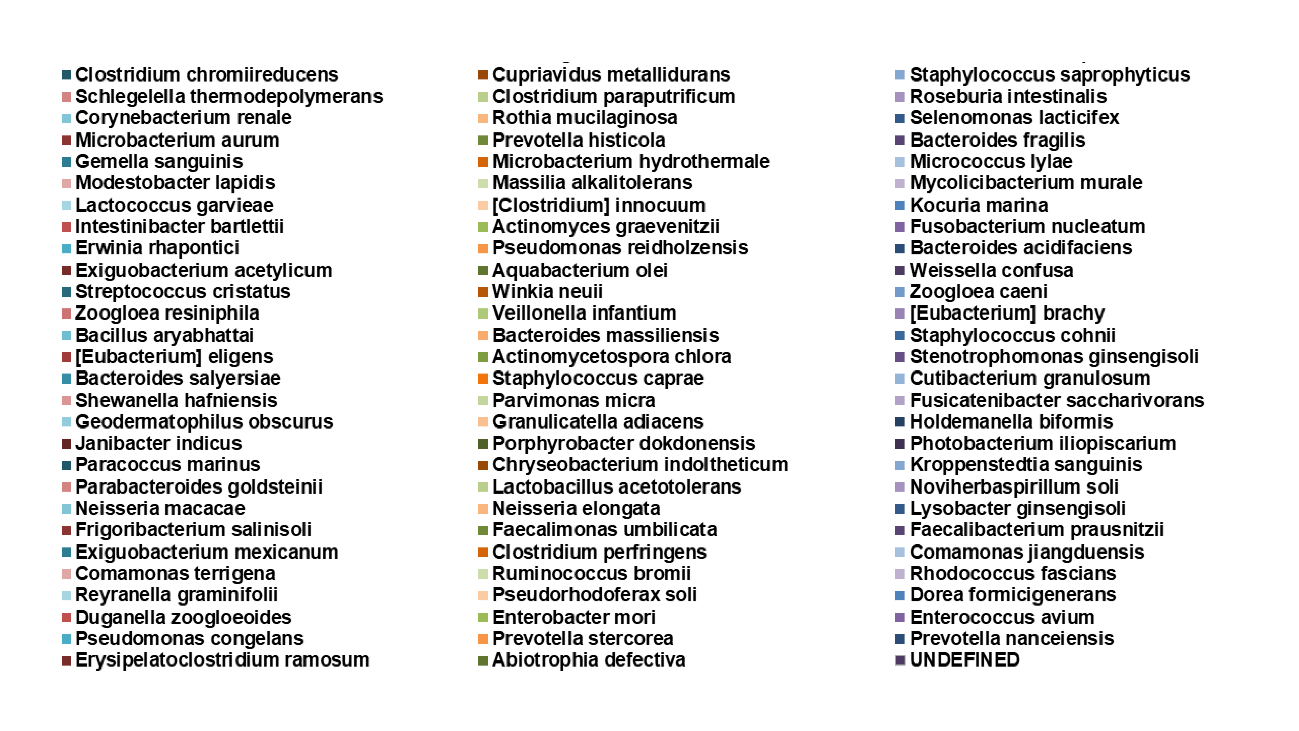
**

**Figure S6. The composition in the skin microbiota at the levels of species**

The relative abundances of skin microbiome at species level in skin samples of the control and IMQ groups. There were significantly changes for fourteen microbes between the two groups (**Table S5**).

**Supplementary Table 1. The gut bacteria that significantly differ between control and IMQ groups at the genus level.**

| Genus | Relative abundance in Control (%) | Relative abundance in IMQ (%) | Mann-Whitney U test |
| --- | --- | --- | --- |
| *Bacteroides* | 2.012 ± 0.627 | 6.429 ± 2.188 | U= 81, P= 0.019 |
| *Parabacteroides* | 0.689 ± 0.131 | 0.040 ± 0.014 | U= 0, P= 0.000 |
| *Staphylococcus* | 0.017 ± 0.005 | 0.081 ± 0.017 | U= 93, P= 0.000 |
| *Faecalimonas* | 0.028 ± 0.006 | 0.010 ± 0.003 | U= 21, P= 0.029 |
| *Alistipes* | 0.030 ± 0.008 | 0.001 ± 0.001 | U= 6, P= 0.000 |

The values are the mean ± S.E.M. (n=10).

**Supplementary Table 2. The gut bacteria that significantly differ between control and IMQ groups at the species level.**

| Species | Relative abundance in Control (%) | Relative abundance in IMQ (%) | Mann-Whitney U test |
| --- | --- | --- | --- |
| *Lactobacillus murinus* | 30.099 ± 4.761 | 10.354 ± 2.291 | U= 12, P= 0.003 |
| *Lactobacillus intestinalis* | 2.700 ± 0.802 | 17.062 ± 3.457 | U= 93, P= 0.000 |
| *Lactobacillus reuteri* | 2.139 ± 0.480 | 7.020 ± 1.166 | U= 86, P= 0.005 |
| *Lactobacillus taiwanensis* | 1.324 ± 0.274 | 2.960 ± 0.402 | U= 86, P= 0.005 |
| *Bacteroides uniformis* | 0.760 ± 0.286 | 3.460 ± 1.379 | U= 79, P= 0.029 |
| *Bacteroides acidifaciens* | 0.980 ± 0.336 | 2.531 ± 0.711 | U= 83, P= 0.011 |
| *Bacteroides sartorii* | 0.105 ± 0.019 | 0.009 ± 0.004 | U= 3.5, P= 0.000 |
| *Staphylococcus lentus* | 0.003 ± 0.002 | 0.079 ± 0.017 | U= 100, P= 0.000 |
| *Parabacteroides distasonis* | 0.047 ± 0.014 | 0.001 ± 0.001 | U= 6.5, P= 0.000 |

The values are the mean ± S.E.M. (n=10).

**Supplementary Table 3. The skin bacteria that significantly differ between control and IMQ groups at the phylum level.**

| Phylum | Relative abundance in Control (%) | Relative abundance in IMQ (%) | Mann-Whitney U test |
| --- | --- | --- | --- |
| *Firmicutes* | 83.270 ± 5.256 | 99.242 ± 0.477 | U= 99, P= 0.000 |
| *Proteobacteria* | 9.636 ± 5.037 | 0.137 ± 0.073 | U= 1, P= 0.000 |
| *Cyanobacteria* | 4.498 ± 0.884 | 0.432 ± 0.333 | U= 3.5, P= 0.000 |
| *Actinobacteria* | 1.520 ± 0.138 | 0.065 ± 0.029 | U= 0, P= 0.000 |
| *Bacteroidetes* | 1.048 ± 0.267 | 0.122 ± 0.051 | U= 5, P= 0.000 |

The values are the mean ± S.E.M. (n=10).

**Supplementary Table 4. The skin bacteria that significantly differ between control and IMQ groups at the genus level.**

| Genus | Relative abundance in Control (%) | Relative abundance in IMQ (%) | Mann-Whitney U test |
| --- | --- | --- | --- |
| *Staphylococcus* | 16.200 ± 1.551 | 98.513 ± 0.914 | U= 100, P= 0.000 |
| *Lactobacillus* | 46.420 ± 4.652 | 0.413 ± 0.287 | U= 0, P= 0.000 |
| *Jeotgalicoccus* | 2.011 ± 0.331 | 0.038 ± 0.034 | U= 0, P= 0.000 |
| *Atopostipes* | 1.637 ± 0.187 | 0.044 ± 0.040 | U= 0, P= 0.000 |
| *Turicibacter* | 1.299 ± 0.373 | 0.009 ± 0.006 | U= 0, P= 0.000 |
| *Corynebacterium* | 0.808 ± 0.102 | 0.020 ± 0.018 | U= 0, P= 0.000 |
| *Enterococcus* | 0.326 ± 0.115 | 0.016 ± 0.009 | U= 3.5, P= 0.000 |
| *Clostridium* | 0.351 ± 0.079 | 0.004 ± 0.002 | U= 0, P= 0.000 |
| *Faecalicatena* | 0.263 ± 0.049 | 0.001 ± 0.001 | U= 0, P= 0.000 |
| *Sphingomonas* | 0.030 ± 0.008 | 0.000 ± 0.000 | U= 5, P= 0.000 |
| *Enterorhabdus* | 0.144 ± 0.023 | 0.001 ± 0.001 | U= 0, P= 0.000 |
| *Acinetobacter* | 0.069 ± 0.015 | 0.002 ± 0.002 | U= 1, P= 0.000 |
| *Sporosarcina* | 0.106 ± 0.024 | 0.002 ± 0.002 | U= 1, P= 0.000 |
| *Acutalibacter* | 0.053 ± 0.011 | 0.000 ± 0.000 | U= 0, P= 0.000 |
| *Parvibacter* | 0.044 ± 0.008 | 0.000 ± 0.000 | U= 5, P= 0.000 |
| *Adlercreutzia* | 0.042 ± 0.011 | 0.000 ± 0.000 | U= 5, P= 0.000 |

The values are the mean ± S.E.M. (n=10).

**Supplementary Table 5. The skin bacteria that significantly differ between control and IMQ groups at the species level.**

| Species | Relative abundance in Control (%) | Relative abundance in IMQ (%) | Mann-Whitney U test |
| --- | --- | --- | --- |
| *Staphylococcus lentus* | 7.138 ± 0.869 | 93.547 ± 1.534 | U= 100, P= 0.000 |
| *Lactobacillus murinus* | 25.643 ± 2.708 | 0.191 ± 0.124 | U= 0, P= 0.000 |
| *Lactobacillus intestinalis* | 9.621 ± 1.180 | 0.118 ± 0.092 | U= 0, P= 0.000 |
| *Lactobacillus reuteri* | 6.543 ± 0.693 | 0.046 ± 0.035 | U= 0, P= 0.000 |
| *Lactobacillus taiwanensis* | 4.021 ± 0.326 | 0.044 ± 0.032 | U= 0, P= 0.000 |
| *Turicibacter sanguinis* | 1.299 ± 0.373 | 0.009 ± 0.006 | U= 0, P= 0.000 |
| *Corynebacterium ammoniagenes* | 0.791 ± 0.103 | 0.020 ± 0.018 | U= 0, P= 0.000 |
| *Atopostipes suicloacalis* | 0.549 ± 0.066 | 0.002 ± 0.001 | U= 0, P= 0.000 |
| *Clostridium disporicum* | 0.348 ± 0.078 | 0.002 ± 0.001 | U= 0, P= 0.000 |
| *Enterococcus faecalis* | 0.068 ± 0.019 | 0.001 ± 0.001 | U= 0, P= 0.000 |
| *Enterorhabdus caecimuris* | 0.058 ± 0.012 | 0.001 ± 0.001 | U= 0, P= 0.000 |
| *Parvibacter caecicola* | 0.044 ± 0.008 | 0.000 ± 0.000 | U= 5, P= 0.000 |
| *Adlercreutzia muris* | 0.042 ± 0.011 | 0.000 ± 0.000 | U= 5, P= 0.000 |
| *Acutalibacter muris* | 0.035 ± 0.010 | 0.000 ± 0.000 | U= 5, P= 0.000 |

The values are the mean ± S.E.M. (n=10).
